# The impact of obesity and endocrine therapy on the prognosis of premenopausal women with hormone receptor‐positive breast cancer: A single‐institute retrospective study

**DOI:** 10.1002/cnr2.1695

**Published:** 2022-08-09

**Authors:** Yukinori Ozaki, Jun Masuda, Akemi Kataoka, Takahiro Kogawa, Tomomi Abe, Hidetomo Morizono, Lina Inagaki, Fumikata Hara, Toshimi Takano, Takayuki Ueno, Shinji Ohno

**Affiliations:** ^1^ Breast Oncology Center The Cancer Institute Hospital of JFCR Tokyo Japan; ^2^ Division of Early Clinical Development for Cancer, Advanced Medical Development Center The Cancer Institute Hospital of JFCR Tokyo Japan

**Keywords:** body mass index, breast cancer, ovarian function suppression, premenopausal

## Abstract

**Background:**

Higher body mass index (BMI) is associated with worse prognosis in pre‐ and postmenopausal patients with breast cancer (BC). However, there is insufficient evidence regarding the optimal adjuvant endocrine therapy for obese premenopausal women with hormone receptor (HR)‐positive BC.

**Aim:**

To evaluate the impact of obesity and adjuvant endocrine therapy on prognosis in premenopausal patients with BC.

**Methods and results:**

We retrospectively reviewed the medical record of premenopausal women who received curative surgery for clinical stage I–III HR‐positive BC from 2007 to 2017. Patients were classified into five groups according to BMI: underweight (UW), normal weight (NW), obese 1 degree (OB1), obese 2 degree (OB2), and obese 3 degree (OB3) categories. The primary analysis was a comparison of BC‐specific survival (BCSS) according to BMI (UW/NW vs. OB1–3) and adjuvant endocrine therapy (with or without ovarian function suppression [OFS]).

Of 13 021 patients, the data of 3380 patients were analyzed. BCSS in OB1–3 patients was significantly worse than that in patients with UW/NW (hazard ratio [HR] 2.37; 95% confidence interval [CI], 1.40–4.02: *p* = .0009). In OB1–3 patients who received tamoxifen (TAM), BCSS was significantly worse than that in UW/NW patients (*p* = .0086); however, a significant difference was not shown in patients who received TAM and OFS (*p* = .0921).

**Conclusion:**

High BMI was associated with worse prognosis in premenopausal patients with HR‐positive BC who received adjuvant TAM. The role of OFS as adjuvant endocrine therapy remains unclear, and further studies are required to explore the adequate management of obese premenopausal patients.

## 1 INTRODUCTION

Obesity (defined by the World Health Organization as a body mass index [BMI] of ≥30 kg/m^2^) has been associated with an increased risk of developing BC,^1^, ^2^ and obesity is associated with worse prognosis of BC in pre‐ and postmenopausal women.^3^, ^4^, ^5^, ^6^, ^7^ Several reports evaluated the impact of obesity on treatment efficacy for BC.^8^, ^9^ However, there is limited evidence regarding the optimal postoperative endocrine therapy for obese premenopausal women with hormone receptor (HR)‐positive BC.

According to a systematic review, obesity has a significant association with overall survival and disease‐free survival in patients with BC, and this study demonstrated that obesity was a poor prognostic factor for recurrence in BC with an integrated effect of 1.12 (95% confidence interval [CI]: 1.06–1.18).^1^ A meta‐analysis of 22 362 patients suggested an association of obesity with BC in Asian premenopausal women, and another population‐based follow‐up study of 1177 premenopausal patients with BC demonstrated that the 5‐year mortality rate (95% CI: 1.6–3.9) of women in the highest quartile of BMI was 2.5 times higher.^10^, ^11^ On the basis of these data, a positive association has consistently been found between high BMI and poor prognosis of BC in pre‐ and postmenopausal women.

The mechanism of poor prognosis of BC in obese patients is not fully understood, but higher estrogen levels associated with obesity is a reasonable explanation of poor prognosis mainly in postmenopausal women with HR‐positive BC. Adipose tissue functions as an endocrine organ, which results a high level of estrogen in obese women.^12^ Although the association of obesity and estradiol levels remains inconsistent in premenopausal women,^13^, ^14^ several studies demonstrated a significant association of BMI with estradiol levels in premenopausal women. One large cohort study showed that higher BMI was associated with a higher concentration of calculated free estradiol, which may be a reliable biomarker reflecting the estrogenic environment in obese premenopausal women.^15^ Thus, a higher level of estrogen in obese premenopausal women is a reasonable mechanism associated with the poor prognosis of HR‐positive BC.

Limited data have been reported regarding the association of obesity and the efficacy of hormone therapy in premenopausal patients with HR‐positive BC.^16^ In a report of the National Surgical Adjuvant Breast and Bowel Project B‐14 cohort, obesity did not affect the efficacy of adjuvant tamoxifen (TAM).^17^ However, the Austrian Breast and Colorectal Cancer Study Group demonstrated that obesity significantly impacts the efficacy of anastrozole and goserelin in premenopausal patients.^18^ These data suggest that the impact of obesity on the efficacy of adjuvant hormone therapy in premenopausal patients is still controversial, and the effect of ovarian function suppression (OFS) with TAM in premenopausal obese patients with HR‐positive BC has not been reported.

We hypothesized that a higher BMI is associated with worse survival outcomes in premenopausal patients with HR‐positive primary BC, and that the impact differs depending on whether patients receive OFS or not. The primary objective of this study was to determine whether higher BMI was associated with worse BC‐specific survival (BCSS) in premenopausal patients with HR‐positive primary BC with or without OFS treatment. Our secondary objective was to evaluate BCSS and overall survival (OS) according to BMI.

## 2 METHODS

### 2.1 Study design and patients

This retrospective study was performed using medical records from female patients who underwent curative surgery for clinical stage I–III HR‐positive primary BC at the Cancer Institute Hospital of the Japanese Foundation for Cancer Research (CIH) from January 2007 to December 2017. This study protocol (approval no. 2018‐1100) was approved by the CIH's independent ethical committee/institutional review board and was carried out in accordance with the Helsinki Declaration. Because of the retrospective nature of this study, informed consent was waived.

The clinicopathological characteristics of the patients included age and menopausal status at the time of diagnosis. Postmenopausal definition was no menstruation within the last year or a previous history of bilateral oophorectomy. BMI was calculated by weight at surgery. Patients received standard treatment according to the guidelines of the Japanese Breast Cancer Society. The inclusion criteria for the analysis were: premenopausal patients who received curative surgery for clinical stage I–III HR‐positive primary BC; and patients who received TAM or TAM and OFS, leuprolide acetate, or goserelin acetate, as adjuvant endocrine therapy. Patients with a history of other invasive cancers and bilateral BC were excluded. Patients who had complete data on baseline weight and BMI before surgery were enrolled in this study. Indication of OFS and the duration of TAM for each premenopausal patient was discussed at an internal conference depending on clinicopathological features, and goserelin acetate or leuprorelin acetate was used for 2–5 years and TAM was used for 5–10 years. Patients were followed at least once a year for the next 10 years after surgery. Access to hospital visitation records, resident registration cards, and permanent domicile data were used to examine subsequent survival. BMI was calculated by dividing the weight in kilograms by the square of the height in meters and categorized using the definition of the Japan Society for the Study of Obesity as follows: underweight (UW), BMI <18.5 kg/m^2^; normal weight (NW), 18.5–24.9 kg/m^2^; obese 1 degree (OB1), 25.0–29.9 kg/m^2^; obese 2 degree (OB2), 30.0–34.9 kg/m^2^; and obese 3 degree (OB3), ≥35 kg/m^2^.

### 2.2 Pathological assessment

Pathological information including nuclear grade, tumor size, lymph node status, and immunohistochemical (IHC) results of estrogen receptor (ER), progesterone receptor (PR), human epidermal growth factor receptor 2 (HER2), and Ki67 labeling index assessed by two independent pathologists were obtained from patients' pathological reports. ER positivity (ER+) and PR positivity (PR+) were defined as 1% or more positive invasive tumor cells showing nuclear staining. Hormone receptor positivity (HR+) was defined as ER+ and/or PR+. According to the 2007 American Society of Clinical Oncology/College of American Pathologists guidelines, HER2 positivity was defined as a HER2/CEP17 FISH ratio of 2.0 and/or an IHC staining score of 3+.^19^ The percentage of Ki67 positivity was calculated for ~1000 cells in hot spots and was divided into three categories: low for 10% or less, intermediate for 10%–30%, and high for 30% or more.^20^

### 2.3 Endpoints and statistical analysis

**FIGURE 1:**
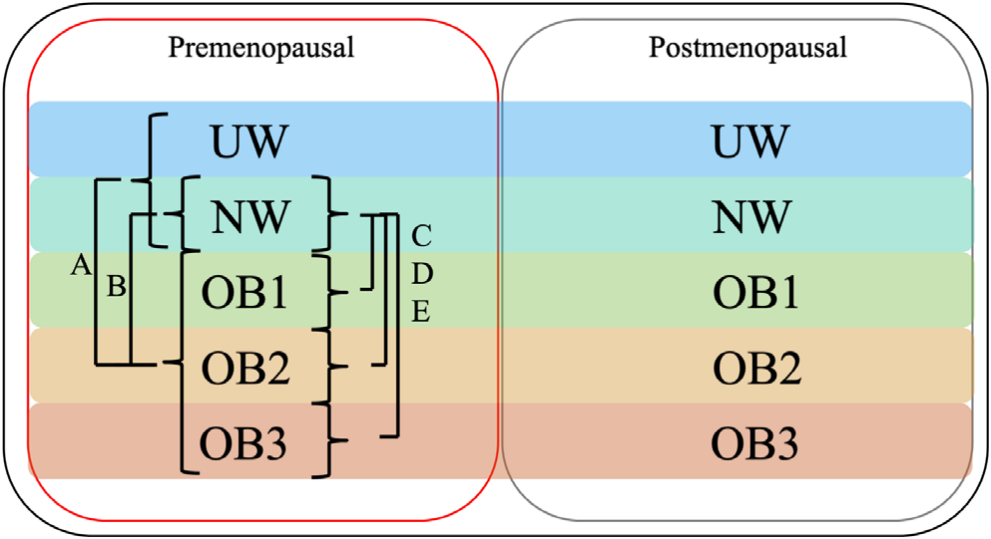
Patient population for survival analysis. Primary analysis of BCSS was performed in UW/NW patients versus OB1–3 patients (A). To evaluate the role of OFS, a subgroup analysis was performed in patients who received TAM without OFS and in patients who received TAM with OFS. The secondary analysis was overall survival. An exploratory analysis was also performed in patients who received TAM versus TAM and OFS in OB1–3. BCSS, breast cancer‐specific survival; NW, normal weight; OB1, obese 1 degree; OB2, obese 2 degree; OB3, obese 3 degree; OFS, ovarian function suppression; TAM, tamoxifen; UW, underweight

The primary endpoint was BCSS, defined as the time interval from the date of curative BC surgery to the date of death from BC. The secondary endpoint was OS, defined as the time interval from the date of BC surgery to the date of death from any cause. The primary analysis was a comparison of BCSS according to BMI (UW/NW vs. OB1–3), using the log‐rank test and the Cox proportional hazards model (Figure 1). A comparison of BCSS according to BMI was conducted in patients who received TAM or in patients who received TAM and OFS, respectively. A propensity matching model was used to evaluate the effect of OFS in OB1–3 patients. A subgroup analysis of OS was performed, and a comparison between NW and OB1–3 was also preplanned. To evaluate the impact of BMI on the prognosis of high‐risk premenopausal patients, a subgroup analysis of stages I and II/III was performed. Logistic regression analysis was used to evaluate the association of patient characteristics and BMI. Univariate models (cross‐tabulation and two‐sided *χ* ^2^ test) were used to evaluate the predictive effect of categorical variables on BMI. The Kaplan–Meier method was used to plot survival curves, which were then compared between groups using the log‐rank test. For clinicopathological variables and survival, hazard ratios and 95% CIs were calculated using univariate and multivariate Cox proportional hazards regression analyses. All analyses were performed using JMP 14 (SAS Institute Inc., Cary, NC, United States) and SAS version 9.4 (SAS Institute Inc.). All reported *p* values were two‐sided, and *p* < .05 was considered statistically significant.

## 3 RESULTS

### 3.1 Patients' characteristics

**FIGURE 2:**
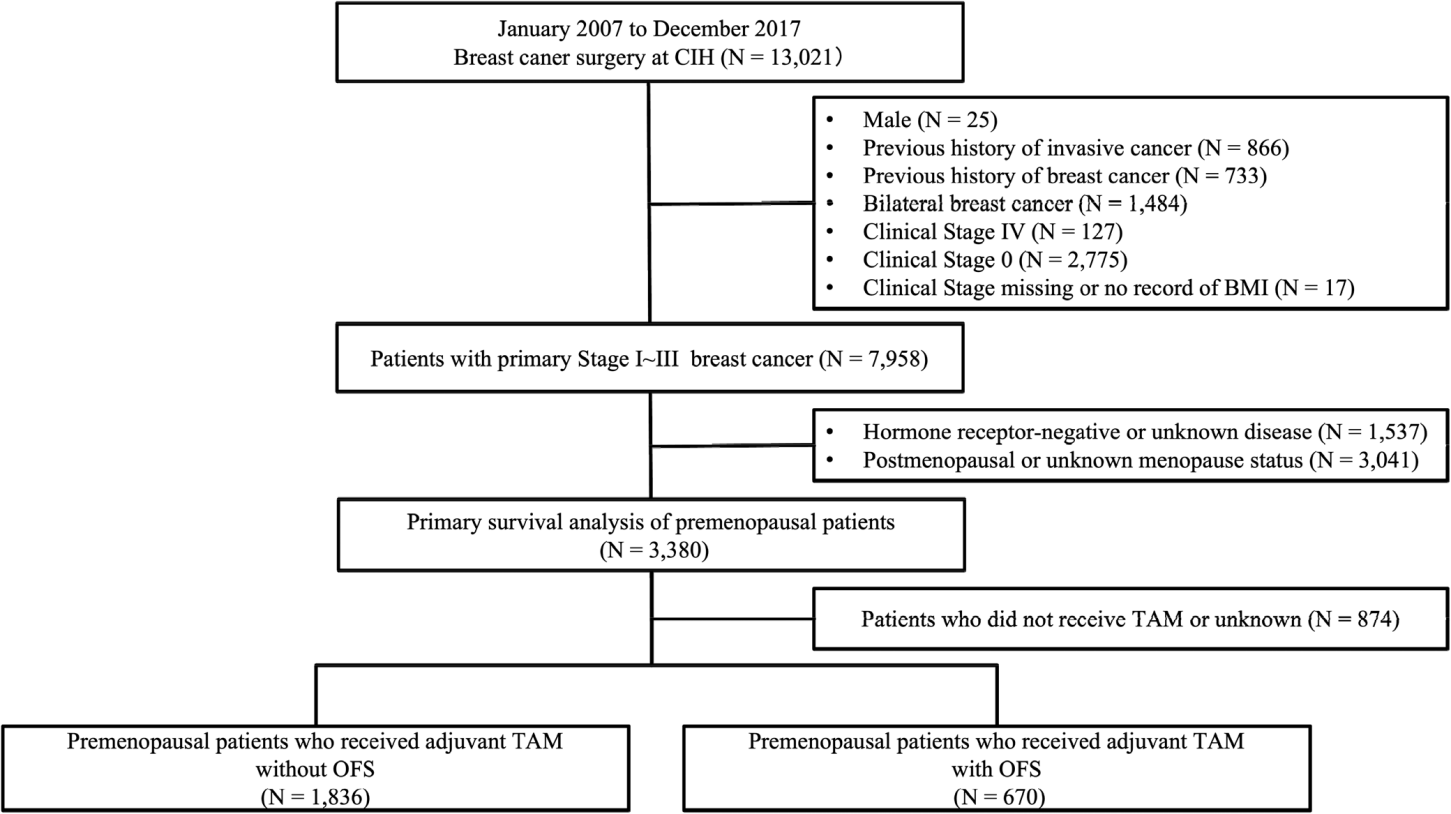
CONSORT diagram. CIH, Cancer Institute Hospital; OFS, ovarian function suppression; TAM, tamoxifen

From January 2007 to December 2017, 13 021 BC surgeries were performed. In total, 3380 premenopausal women with primary, HR‐positive, stage I, II, or III BC were included in the survival analysis, as shown in a CONSORT diagram (Figure 2). The primary analysis was a survival analysis in premenopausal patients with HR‐positive BC, which included patients who received TAM without OFS (*n* = 1836) or with OFS (*N* = 670). The median follow‐up duration of the patients was 5.9 years (range, 0.2–12.9 years). Patients' characteristics are shown in Table 1.

**TABLE 1:** Patients' characteristics

Characteristics	*N* = 3380 (%)
Age (years)	
Median	45 (range: 18–58)
Body mass index	
UW (<18.5 kg/m^2^)	404 (12)
NW (18.5–24.9 kg/m^2^)	2482 (73)
OB1 (25.0–29.9 kg/m^2^)	399 (12)
OB2 (30.0–34.9 kg/m^2^)	78 (2)
OB3 (≥35 kg/m^2^)	17 (1)
Clinical stage	
I	1990 (59)
II	1214 (36)
III	176 (5)
HER2 status	
HER2–	2937 (86)
HER2+	287 (9)
HER2 unknown	156 (5)
Number of metastatic lymph nodes	
0	2214 (66)
1–3	886 (26)
4–9	218 (6)
≥10	62 (2)
Ki‐67^a^ (*N* = 865)	
Low (<10%)	298 (34)
Intermediate ≥ 10% and <30%	338 (39)
High (≥30%)	229 (26)
Nuclear grade (*N* = 2562)	
1	1149 (45)
2	1124 (44)
3	289 (11)
Neo‐adjuvant/adjuvant chemotherapy	
Yes	1744 (52)
No	1636 (48)
Tamoxifen (*N* = 2995)	
Yes	2506 (84)
No	489 (16)
Aromatase inhibitor (*N* = 2995)	
Yes	322 (11)
No	2673 (89)
OFS (*N* = 2995)	
Yes	680 (23)
No	2315 (77)

Abbreviations: HER2, human epidermal growth factor receptor 2; HR, hormone receptor; NW, normal weight; OB1, obese 1 degree; OB2, obese 2 degree; OB3, obese 3 degree; OFS, ovarian function suppression; UW, underweight.

^a^ The percentage of Ki67 positivity was calculated for ~1000 cells in hot spots.

The association of patient characteristics with the BMI category is shown in Table 2. Logistic regression analysis showed a significant association of BMI with age (≤40 or >40 years), stage, and number of lymph nodes. Patients with a higher BMI were associated with a higher stage of BC (I, II, or III) (*p* < .0001) and a greater number of lymph node metastases (0, 1–3, 4–9, or ≥10) (*p* = .0078), as shown in Table 2. Higher nuclear grade (1 vs. 2 and 3) was significantly associated with OB1–3 compared with UW/NW (*p* = .0366). Ki67 index (low vs. intermediate and high) tended to be associated with a higher BMI without statistical significance (*p* = .1625).

**TABLE 2:** Patients' characteristics by BMI category

Characteristics	BMI category *N* = 3380 (%)					
	LW <18.5 kg/m^2^	NW 18.5–24.9 kg/m^2^	OB1 25.0–29.9 kg/m^2^	OB2 30.0–34.9 kg/m^2^	OB3 ≥35 kg/m^2^	
All	384 (12)	2363 (73)	386 (12)	74 (2)	17 (1)	
Age						
≤40	115 (15)	582 (75)	62 (8)	12 (2)	1 (0)	*p* < .0001
>40	289 (11)	1900 (73)	337 (13)	66 (3)	16 (1)	
HER2 status (*N* = 3224)						
HER2‐	354 (12)	2149 (73)	351 (12)	67 (2)	16 (1)	*p* = .9233
HER2+	30 (10)	214 (75)	35 (12)	7 (2)	1 (0)	
cStage						
I	251 (13)	1502 (75)	193 (10)	35 (2)	9 (0)	*p* < .0001
II	135 (11)	861 (71)	172 (14)	38 (3)	8 (1)	
III	18 (10)	119 (68)	34 (19)	5 (3)	0	
Number of lymph nodes						
0	292 (13)	1627 (73)	243 (11)	42 (2)	10 (0)	*p* = .0466
1–3	87 (10)	654 (74)	113 (13)	27 (3)	5 (1)	
4–9	18 (8)	160 (73)	30 (14)	8 (4)	2 (1)	
≥10	7 (11)	41 (66)	13 (21)	1 (2)	0	
Ki67 index (*N* = 865)						
Low	35 (12)	218 (73)	40 (13)	5 (2)	0	*p* = .4737
Intermediate	41 (12)	239 (71)	47 (14)	8 (2)	3 (1)	
High	26 (11)	165 (72)	33 (14)	5 (2)	0	
NG (*N* = 2562)						
1	146 (13)	859 (75)	118 (10)	23 (2)	3 (0)	*p* = .2768
2	131 (12)	818 (73)	142 (13)	25 (2)	8 (1)	
3	36 (12)	222 (77)	23 (8)	7 (2)	1 (0)	

Abbreviations: BMI, body mass index; HER2, human epidermal growth factor receptor 2; HR, hormone receptor; NG, nuclear grade; NW, normal weight; OB1, obese 1 degree; OB2, obese 2 degree; OB3, obese 3 degree; UW, underweight.

**FIGURE 3:**
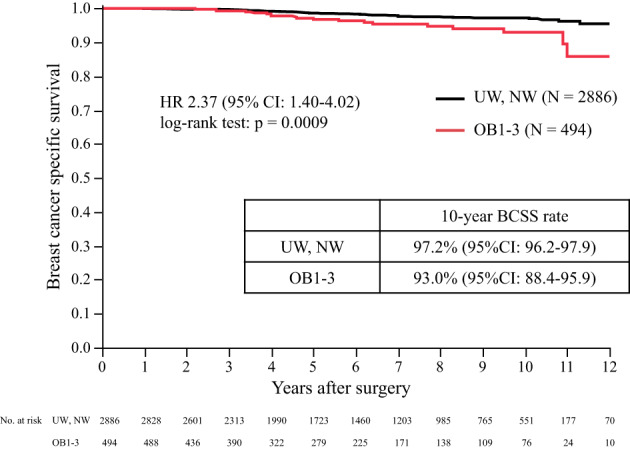
Breast cancer‐specific survival by BMI. Breast cancer‐specific survival in premenopausal patients by BMI (UW, NW vs. OB1–3). BCSS, breast cancer‐specific survival; BMI, body mass index; CI, confidence interval; HR, hazard ratio; NW, normal weight; OB1, obese 1 degree; OB2, obese 2 degree; OB3, obese 3 degree; UW, underweight

**FIGURE 4:**
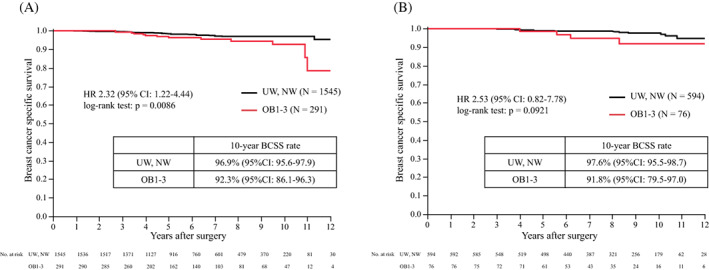
Breast cancer‐specific survival. (A) Breast cancer‐specific survival in premenopausal patients who received TAM without OFS. (B) Breast cancer‐specific‐survival in premenopausal patients who received TAM with OFS. BCSS, breast cancer‐specific survival; CI, confidence interval; HR, hazard ratio; NW, normal weight; OB1, obese 1 degree; OB2, obese 2 degree; OB3, obese 3 degree; OFS, ovarian function suppression; TAM, tamoxifen; UW, underweight

**FIGURE 5:**
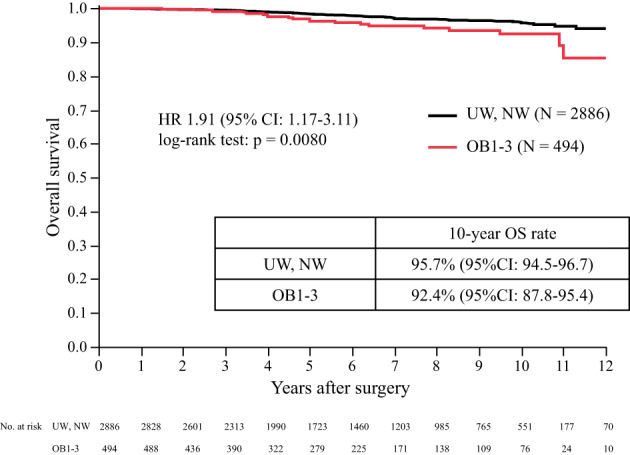
Overall survival by BMI. Overall survival in premenopausal patients by BMI (UW, NW vs. OB1–3). BCSS, breast cancer‐specific survival; BMI, body mass index; CI, confidence interval; HR, hazard ratio; NW, normal weight; OB1, obese 1 degree; OB2, obese 2 degree; OB3, obese 3 degree; UW, underweight

The primary analysis of BCSS in premenopausal patients by the BMI subgroup is shown in Figure 3. The 10‐year BCSS of UW/NW was 97.2% (95% CI: 96.2–97.9) and that of OB1–3 was 93.0% (95% CI: 88.4–95.9). BCSS with OB1–3 was significantly worse than that in patients with UW/NW (hazard ratio [HR] 2.37; 95% CI, 1.40–4.02; *p* = .0009). To evaluate the effect of OFS, BCSS analysis was performed in subgroups with or without OFS. Figure 4A shows that the BCSS of OB1–3 in patients who received TAM without OFS was significantly worse than that of UW/NW (10‐year BCSS 92.3% vs. 96.9%, HR 2.32, 95% CI: 1.22–4.44, *p* = .0086). However, the BCSS of OB1–3 in patients who received TAM with OFS was not significantly different from that of UW/NW (10‐year BCSS 91.8% vs. 97.6%, HR 2.53, 95% CI: 0.82–7.78, *p* = .0921). In the OB1–3 patients, the BCSS of patients who received TAM and OFS was not significantly different from that in patients who received TAM without OFS (Figure S1). High‐risk patients were included in the TAM with OFS subgroup and not in the TAM without OFS subgroup. To adjust the patient background factors, a matching model with a propensity score, calculated by patient background including age, stage, HER2 status, lymph node status, nuclear grade (NG), and Ki67, was used and did not show a significant difference between the BCSS of patients with TAM and that of patients with TAM and OFS (*n* = 106) (*p* = .1266) (Figure S2). OS, a secondary endpoint, showed that OB1–3 was associated with a significantly poorer prognosis than UW/NW (10‐year OS 92.4% vs. 95.7%, HR 1.91, 95% CI: 1.17–3.11, *p* = .0080) in Figure 5.

To evaluate the consistency of the prognostic effect of OB1–3 compared with that of NW, we analyzed BCSS between NW patients and OB1, OB2, OB3, and OB1–3 patients (Figure 1). The 10‐year BCSS rates of NW, OB1, OB2, OB3, and OB1–3 patients were 97.1% (95% CI: 96.0–98.0), 94.2% (95% CI: 89.6–96.8), 88.3% (95% CI: 62.2–97.2), 80.0% (95% CI: 45.9–95.0), and 92.4% (95% CI: 94.5–96.7), respectively (Figure S3). BCSS of OB1–3 was significantly worse than that of NW patients (HR 2.34, 95% CI: 1.37–4.00, *p* = .0014). BCSS of OB1, OB2, and OB3 patients was significantly worse than that of NW patients (*p* = .0304, *p* = .0141, and *p* = .0007, respectively), which was consistent with the results of the primary analysis. The OS analysis by BMI categories showed that the 10‐year OS rates of NW, OB1, OB2, OB3, and OB1–3 patients were 95.4% (95% CI: 93.8–96.7), 93.0% (95% CI: 87.7–96.2), 86.0% (95% CI: 56.8–96.7), 85.7% (95%CI: 41.9–98.0), and 92.4% (95% CI: 87.8–95.4), respectively (Figure S4). OS of OB1–3 patients was significantly different from that of NW patients (HR 2.94, 95% CI: 1.18–3.19, *p* = .0079) (data not shown), which was consistent with the OS analysis result in Figure 5. BCSS of OB1–3 in patients who received TAM without OFS was significantly worse than that of NW (10‐year BCSS 92.7% vs. 96.8%, HR 2.28, 95% CI: 1.18–4.42, *p* = .00122); however, the difference was not significant in patients who received TAM with OFS (10‐year BCSS 91.8% vs. 97.3%, HR 2.22, 95% CI: 0.72–6.82, *p* = .1519), which was consistent with the result in Figure 4. To evaluate the impact of BMI on the prognosis of high‐risk premenopausal patients, a subgroup analysis according to stage was performed. In the stage II/III subgroup, BCSS in only OB3 patients was significantly worse than that in NW patients (*p* < .0001). In the stage I subgroup, BCSS in OB1 and OB3 was not significantly different from that in NW patients; however, BCSS was significantly worse in OB2 patients than in NW patients (*p* = .0055).

To evaluate the prognostic impact of BMI and other factors, univariate analyses were performed for BMI, stage, lymph node status, HER2 status, NG, Ki67, and chemotherapy. Univariate analysis showed that OB1–3 versus UW/NW (*p* = .0109), stage II/III versus I (*p* < .0001), Ki67 intermediate–high versus low (*p* < .0001), NG 2–3 versus 1 (*p* < .0001), chemotherapy yes versus no (*p* < .0001), and lymph node‐positive versus ‐negative (*p* < .0001) were poor prognostic factors for BCSS. To evaluate the prognostic impact of BMI adjusted for other factors, multivariate analyses were performed. OB1–3 was not a significant prognostic factor (*p* = .7140); however, stage II/III versus I (*p* = .0291), NG 2–3 versus 1 (*p* = .0032), and Ki67 intermediate–high versus low (*p* = .0082) were independent prognostic factors, as shown in Table 3. In the subgroup of stage II/III patients, multivariate analysis showed that OB3 (*p* = .0162) was a significant prognostic factor in addition to lymph node status (*p* = .0084) compared with NW in premenopausal women (Table S1). Multivariate analysis of OS did not show a significant prognostic factor (Table S2).

**TABLE 3:** Multivariate analysis of prognostic factors for BCSS in premenopausal patients

Prognostic factor	Adjusted HR	95% confidential interval	*p* Value
OB1‐3 versus UW/NW	0.80	0.23–2.75	.7140
Stage II/III versus I	2.87	1.08–7.62	.0291
NG 2/3 versus 1	6.18	1.40–27.4	.0032
Ki67 int./high versus low	0.54	0.12–2.49	.0082
Chemotherapy yes versus no	1.03	0.33–3.22	.9585

Abbreviations: BCSS, breast cancer‐specific survival; HR, hazard ratio; NG, nuclear grade; NW, normal weight; OB1, obese 1 degree; OB2, obese 2 degree; OB3, obese 3 degree; UW, underweight.

## 4 DISCUSSION

The present study examined the association of BMI at surgery and the prognosis of premenopausal patients with HR‐positive BC and demonstrated that premenopausal patients with OB1–3 had a worse prognosis than those with UW/NW. Interestingly, the prognostic impact of obesity was maintained when adjuvant endocrine therapy was TAM without OFS, but the prognosis was not significantly different in OB1–3 patients who received TAM with OFS compared with that in UW/NW patients. The role of OFS as adjuvant endocrine therapy in obese premenopausal patients with HR‐positive BC remains unclear, and further research is warranted to investigate the optimal adjuvant therapy in this population.

The mechanism of poor prognosis of BC in obese premenopausal patients remains unclear. A previous report showed that BMI was positively associated with calculated free estradiol in the luteal phase.^15^ Another report demonstrated that a 16%–24% increase in age‐adjusted estradiol was observed in association with obesity throughout the menstrual cycle.^21^ We could not evaluate the association with the poor prognosis of obese premenopausal patients and estradiol levels because of the lack of data, which is a limitation of this study.

The Austrian Breast and Colorectal Cancer Study Group evaluated the influence of BMI on the efficacy of adjuvant endocrine therapy in premenopausal patients. This study showed that overweight premenopausal patients who received OFS and aromatase inhibitor (AI) had a significantly increased risk of disease recurrence (HR 1.60, 95% CI: 1.06–2.41) compared with normal weight patients. In a comparison of AI and TAM, overweight patients treated with AI had a 49% increased risk of disease recurrence compared with those treated with TAM (*p* = .08).^18^ These data suggested that obesity significantly impacts the efficacy of AI plus OFS in premenopausal patients with BC, and that this impact differs between the endocrine treatment agents. Our current study first evaluated the impact of obesity on prognosis between TAM and TAM plus OFS in premenopausal patients, and further research is required to investigate the optimal adjuvant endocrine therapy in this population.

To evaluate the efficacy of OFS in OB1–3 patients, we performed an exploratory analysis, which showed that BCSS in premenopausal OB1–3 patients who received TAM and OFS was not significantly better than that in patients who received TAM. However, the number of patients was small, and a single event was noted only in the matching model analysis. In this cohort, 59% of patients were stage I and 66% were lymph node‐negative, suggesting that the majority were low‐risk patients, which resulted in few events. A large population with a high risk of recurrence in a randomized clinical trial is required to clearly detect the benefit of adding OFS to TAM as adjuvant endocrine therapy.

We observed a significant association of BMI with age (≤40 or >40), stage (I, II, or III), number of lymph nodes (0, 1–3, 4–9, or ≥10) and nuclear grade (1–3, or). Patients with a higher BMI had a higher stage of BC and a greater number of lymph node metastases, which was consistent with a previous report.^22^ Interestingly, higher nuclear grade was associated with obesity, which suggested that obesity was associated with poor prognosis regarding biological effects and not only late diagnosis.

The molecular mechanisms by which obesity contributes to poor outcomes in BC patients are unclear, but there are several hypotheses. First, a higher level of estrogens is associated with poor prognosis of BC. In postmenopausal patients, estrogens are synthesized from androgens via augmented aromatization of androstenedione in peripheral adipose tissue; hence, these obese patients produce more estrogen.^23^, ^24^ In premenopausal patients, increased estradiol was observed in association with obesity throughout the menstrual cycle. Second, obesity has been related to the increased level of inflammatory cytokines such as interleukin‐1, interleukin‐6, interleukin‐8, and tumor necrosis factor 1, which induce the activation of growth pathways including angiogenesis, macrophage influx, and anti‐apoptotic pathways.^25^, ^26^ Third, high BMI increases the circulating levels of insulin and insulin‐like growth factor 1, promoting cell proliferation, tumor initiation, tumor growth, tissue invasion, and metastatic progression.^25^

The recurrence rate of patients with HR‐positive BC remains virtually constant for up to 20 years after diagnosis.^27^ Thus, the late recurrence of patients is an urgent issue. Of interest, the BCSS curves in this study separated at 5 years from surgery and even more after 10 years, suggesting that obesity affects prognosis after completion of adjuvant endocrine therapy and is associated with worse BCSS (Figure 3). In our practice, patients with positive lymph nodes are treated with TAM for 10 years or with sequential treatment of TAM with anastrozole over a total of 10 years, while patients with negative lymph nodes are treated with TAM for only 5 years. For these obese patients, 10 years of adjuvant endocrine therapy may contribute to a better prognosis than 5 years of treatment, although this was not demonstrated in this study. The duration of OFS was 2–5 years, determined according to age, lymph node status, and adjuvant chemotherapy.

The current study had several limitations. First, it was a retrospective study at a single institution. Second, there were no data on the actual completion rate or the duration of endocrine therapy and adjuvant radiotherapy. Third, because the BMI data of the current study were only available at the time of surgery for BC, the relationship between weight change and prognosis could not be examined. Additionally, the BMI data of the patients who received neoadjuvant chemotherapy might be affected by the treatment. Finally, chemotherapy‐induced amenorrhea and serum estradiol concentration data were not available for our cohort.

In conclusion, high BMI was associated with worse prognosis in premenopausal patients with HR‐positive BC who received adjuvant TAM. The role of OFS as adjuvant endocrine therapy in this population remains unclear, and further studies are required to explore the adequate management of obese premenopausal patients.

## AUTHOR CONTRIBUTIONS

**Yukinori Ozaki:** Conceptualization (lead); data curation (lead); formal analysis (lead); investigation (lead); methodology (lead); project administration (equal); resources (lead); writing – original draft (lead); writing – review and editing (lead). **Jun Masuda:** Conceptualization (lead); data curation (lead); formal analysis (lead); investigation (equal); methodology (equal); project administration (equal); software (equal); validation (equal); writing – original draft (lead); writing – review and editing (lead). **Akemi Kataoka:** Conceptualization (lead); data curation (lead); formal analysis (lead); investigation (lead); methodology (equal); project administration (equal); resources (lead); writing – original draft (equal); writing – review and editing (equal). **Takahiro Kogawa:** Conceptualization (equal); formal analysis (equal); methodology (equal); project administration (equal); writing – original draft (equal); writing – review and editing (equal). **Tomomi Abe:** Conceptualization (equal); project administration (equal); writing – original draft (supporting); writing – review and editing (supporting). **Hidetomo Morizono:** Conceptualization (equal); data curation (equal); investigation (equal); writing – original draft (supporting). **Lina Inagaki:** Conceptualization (equal); data curation (equal); investigation (equal); project administration (equal); writing – original draft (supporting). **Fumikata Hara:** Conceptualization (equal); data curation (equal); supervision (equal); writing – original draft (equal); writing – review and editing (equal). **Toshimi Takano:** Conceptualization (equal); data curation (equal); investigation (equal); project administration (equal); supervision (equal); writing – original draft (supporting). **Takayuki Ueno:** Conceptualization (equal); data curation (equal); formal analysis (equal); funding acquisition (lead); project administration (equal); supervision (equal); writing – original draft (equal); writing – review and editing (lead). **Shinji Ohno:** Conceptualization (equal); data curation (equal); formal analysis (equal); funding acquisition (lead); project administration (equal); supervision (lead); writing – original draft (equal).

## CONFLICT OF INTEREST

Yukinori Ozaki received honoraria from Daiichi‐sankyo and Chugai. Akemi Kataoka received honoraria from Pfizer and Artnature Inc. Takahiro Kogawa received honoraria from Daiichi‐Sankyo, Chugai, and Eli Lilly, and research funding from Eli Lilly. Fumikata Hara received speakers' bureaus from Pfizer, Kirin, Eli Lilly, Chugai, Daiichi‐Sankyo and Taiho. Toshimi Takano received honoraria from Daiichi‐Sankyo, Chugai, Kyowa Kirin, Eisai, Pfizer, Eli Lilly, and Celltrion, and research funding from Daiichi‐Sankyo, Chugai, Eisai, Ono, and MSD. Takayuki Ueno received payment or honoraria from Chugai Pharmaceutical Co., Ltd. Eisai Co., Ltd, AstraZeneca, and Novartis Pharma, and Kyowa Kirin. Shinji Ohno received speakers' bureaus from AstraZeneca, Pfizer, Chugai, Eisai, Lilly, and contracted research from Eisai and Taiho. None of the other authors has a relevant conflict of interest in this study.

## ETHICS STATEMENT

This study protocol (approval no. 2018‐1100) was approved by the CIH's independent ethical committee/institutional review board and was carried out in accordance with the Helsinki Declaration.

## PATIENT CONSENT STATEMENT

Informed consent was exempted because of the retrospective format of this study.

## Supporting information


**FIGURE S1 Breast cancer specific survival in OB1‐3 premenopausal patients.** Abbreviations: BCSS, breast cancer specific survival; UW, underweight; NW, normal weight; OB1, obese 1 degree; OB2, obese 2 degree; OB3, obese 3 degree; HR, hazard ratio; TAM, tamoxifen; OFS, ovarian function suppression; CI, confidence interval
**FIGURE S2: Breast cancer specific survival in OB1‐3 patients with a propensity matching model.** Abbreviations: OFS, ovarian function suppression; CI, confidence interval
**FIGURE S3: Breast cancer specific survival by BMI (UW, NW, OB1, OB2 and OB3).** Abbreviations: BMI, body mass index; BCSS, breast cancer specific survival; UW, underweight; NW, normal weight; OB1, obese 1 degree; OB2, obese 2 degree; OB3, obese 3 degree; CI, confidence interval
**FIGURE S4. Overall survival by BMI (UW, NW, OB1, OB2 and OB3).** Abbreviations: BMI, body mass index; OS, overall survival; UW, underweight; NW, normal weight; OB1, obese 1 degree; OB2, obese 2 degree; OB3, obese 3 degree; CI, confidence interval

**TABLE S1** Multivariate analysis of prognostic factors for BCSS in NW and OB3 premenopausal patients with stage II/III breast cancer
**TABLE S2**. Multivariate analysis of prognostic factors for OS in premenopausal patients

## Data Availability

The datasets generated during and/or analyzed during the current study are available from the corresponding author on reasonable request.
