# Supplementary material for: The impact of obesity and endocrine therapy on the prognosis of premenopausal women with hormone receptor‐positive breast cancer: A single‐institute retrospective study
Source: Cancer Rep (Hoboken). 2022 Aug 9;6(2):e1695. doi: 10.1002/cnr2.1695 (PMC9940008; doi:10.1002/cnr2.1695)
Supplement: Supplementary file 1 — FIGURE S1 Breast cancer specific survival in OB1‐3 premenopausal patients. Abbreviations: BCSS, breast cancer specific survival; UW, underweight; NW, normal weight; OB1, obese 1 degree; OB2, obese 2 degree; OB3, obese 3 degree; HR, hazard ratio; TAM, tamoxifen; OFS, ovarian function suppression; CI, confidence interval FIGURE S2: Breast cancer specific survival in OB1‐3 patients with a propensity matching model. Abbreviations: OFS, ovarian function suppression; CI, confidence interval FIGURE S3: Breast cancer specific survival by BMI (UW, NW, OB1, OB2 and OB3). Abbreviations: BMI, body mass index; BCSS, breast cancer specific survival; UW, underweight; NW, normal weight; OB1, obese 1 degree; OB2, obese 2 degree; OB3, obese 3 degree; CI, confidence interval FIGURE S4. Overall survival by BMI (UW, NW, OB1, OB2 and OB3). Abbreviations: BMI, body mass index; OS, overall survival; UW, underweight; NW, normal weight; OB1, obese 1 degree; OB2, obese 2 degree; OB3, obese 3 degree; CI, confidence interval [file CNR2-6-e1695-s002.pptx]

## Slide 1
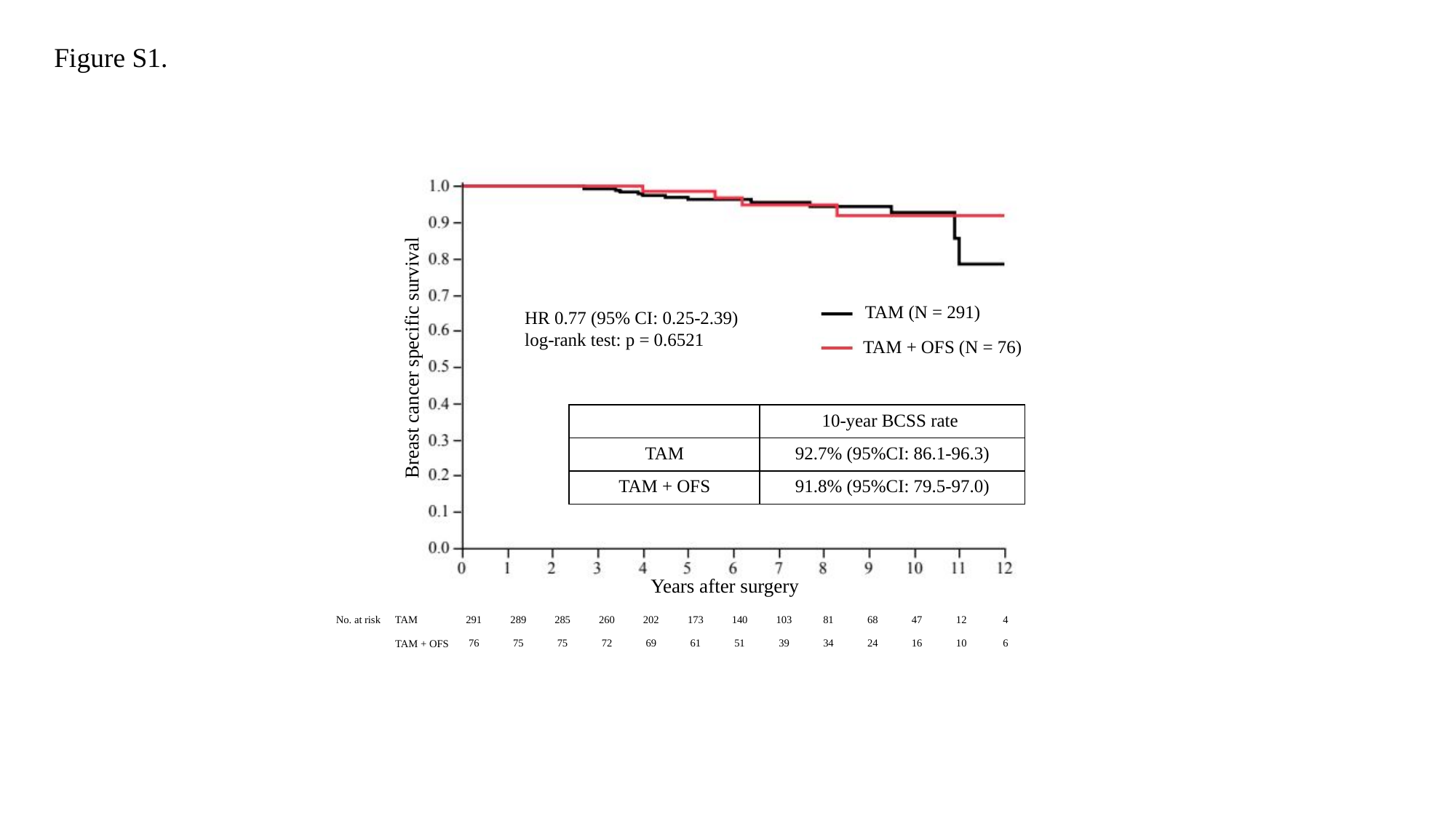

Figure S1.
TAM (N = 291)
TAM + OFS (N = 76)
HR 0.77 (95% CI: 0.25-2.39)
log-rank test: p = 0.6521
Breast cancer specific survival
| | 10-year BCSS rate |
| --- | --- |
| TAM | 92.7% (95%CI: 86.1-96.3) |
| TAM + OFS | 91.8% (95%CI: 79.5-97.0) |
Years after surgery
No. at risk
TAM
| 291 | 289 | 285 | 260 | 202 | 173 | 140 | 103 | 81 | 68 | 47 | 12 | 4 |
| --- | --- | --- | --- | --- | --- | --- | --- | --- | --- | --- | --- | --- |
| 76 | 75 | 75 | 72 | 69 | 61 | 51 | 39 | 34 | 24 | 16 | 10 | 6 |
TAM + OFS

## Slide 2
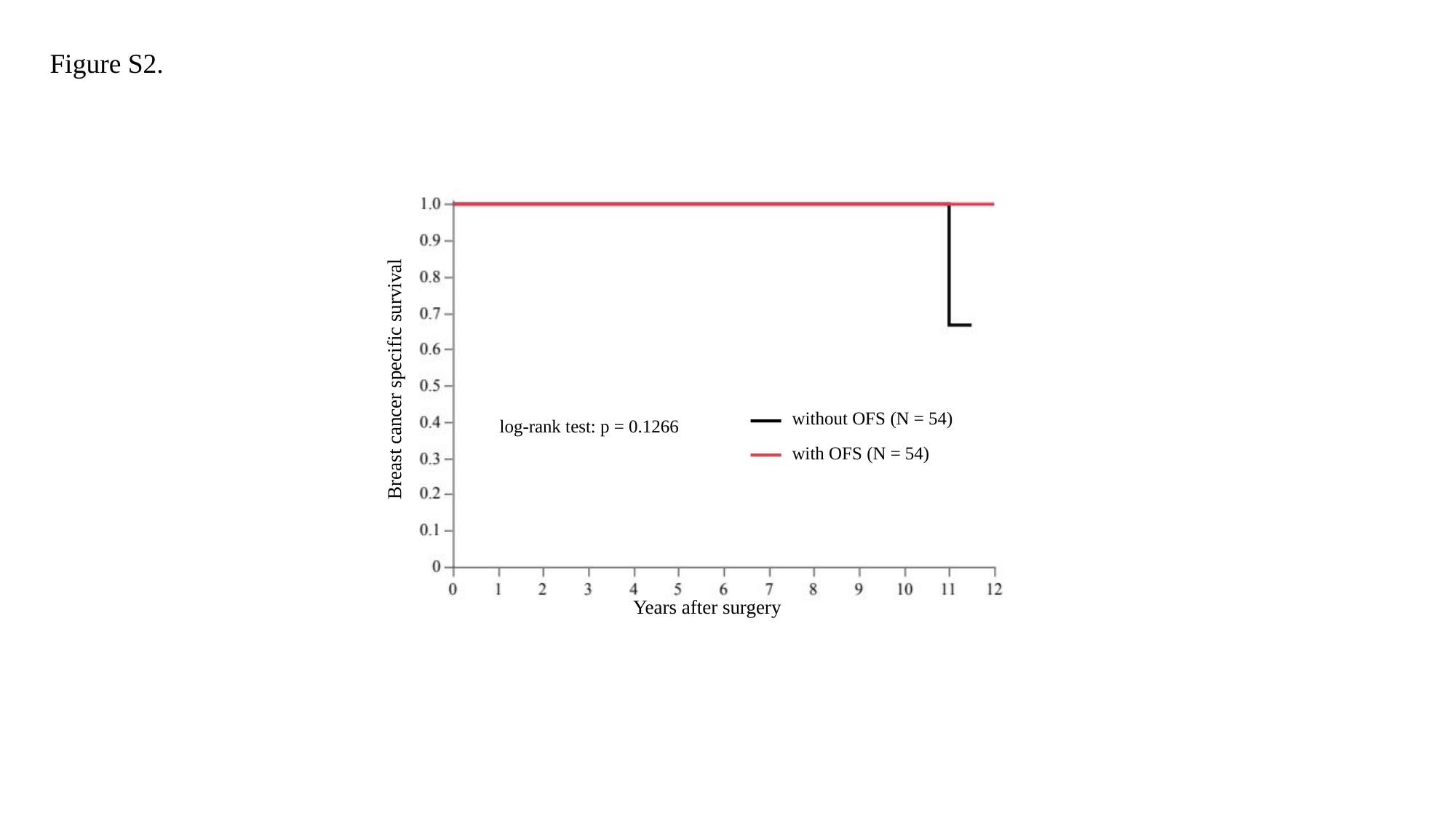

Figure S2.
Breast cancer specific survival
without OFS (N = 54)
with OFS (N = 54)
log-rank test: p = 0.1266
Years after surgery

## Slide 3
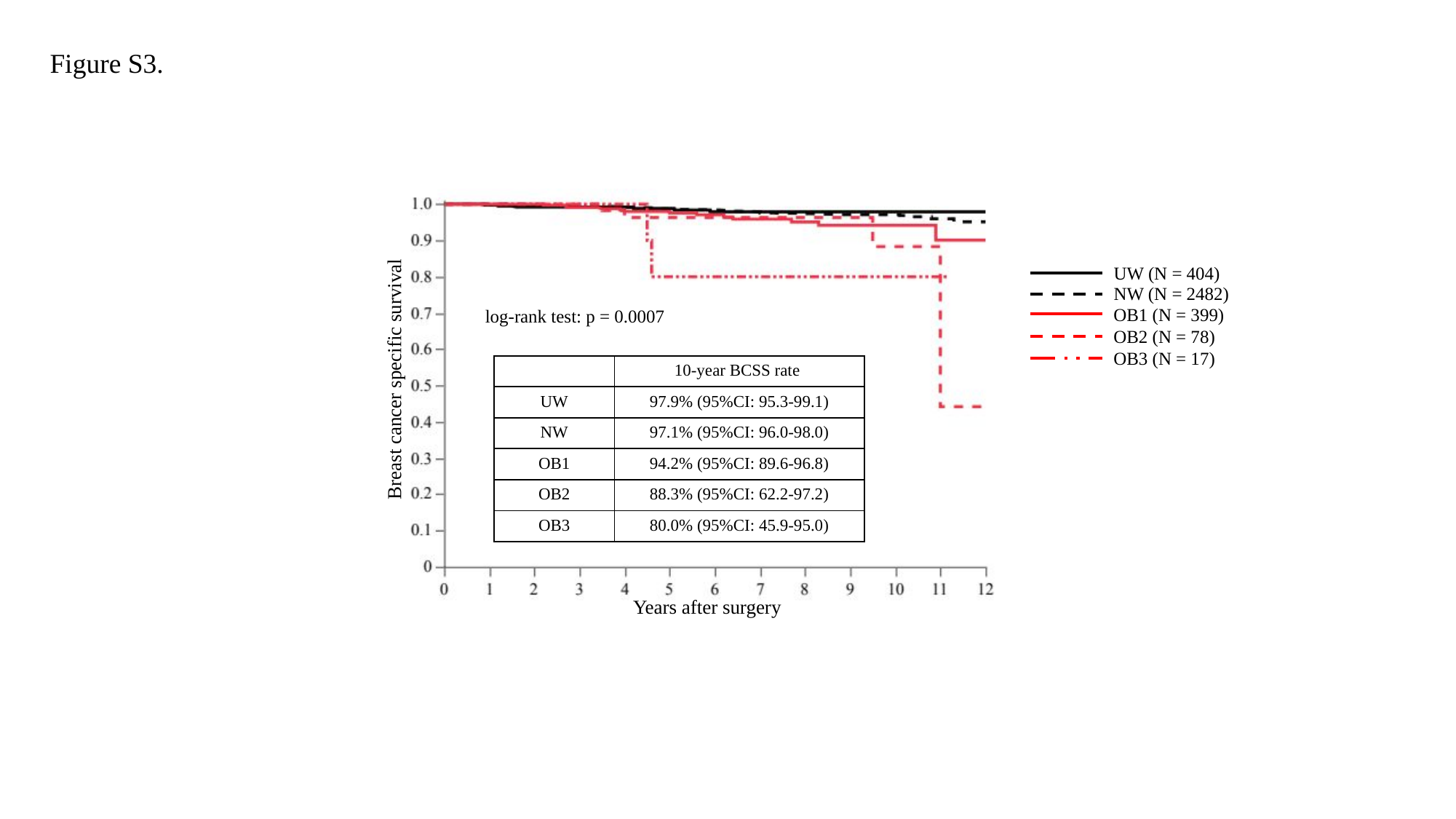

Figure S3.
UW (N = 404)
NW (N = 2482)
OB1 (N = 399)
OB2 (N = 78)
OB3 (N = 17)
log-rank test: p = 0.0007
| | 10-year BCSS rate |
| --- | --- |
| UW | 97.9% (95%CI: 95.3-99.1) |
| NW | 97.1% (95%CI: 96.0-98.0) |
| OB1 | 94.2% (95%CI: 89.6-96.8) |
| OB2 | 88.3% (95%CI: 62.2-97.2) |
| OB3 | 80.0% (95%CI: 45.9-95.0) |
Breast cancer specific survival
Years after surgery

## Slide 4
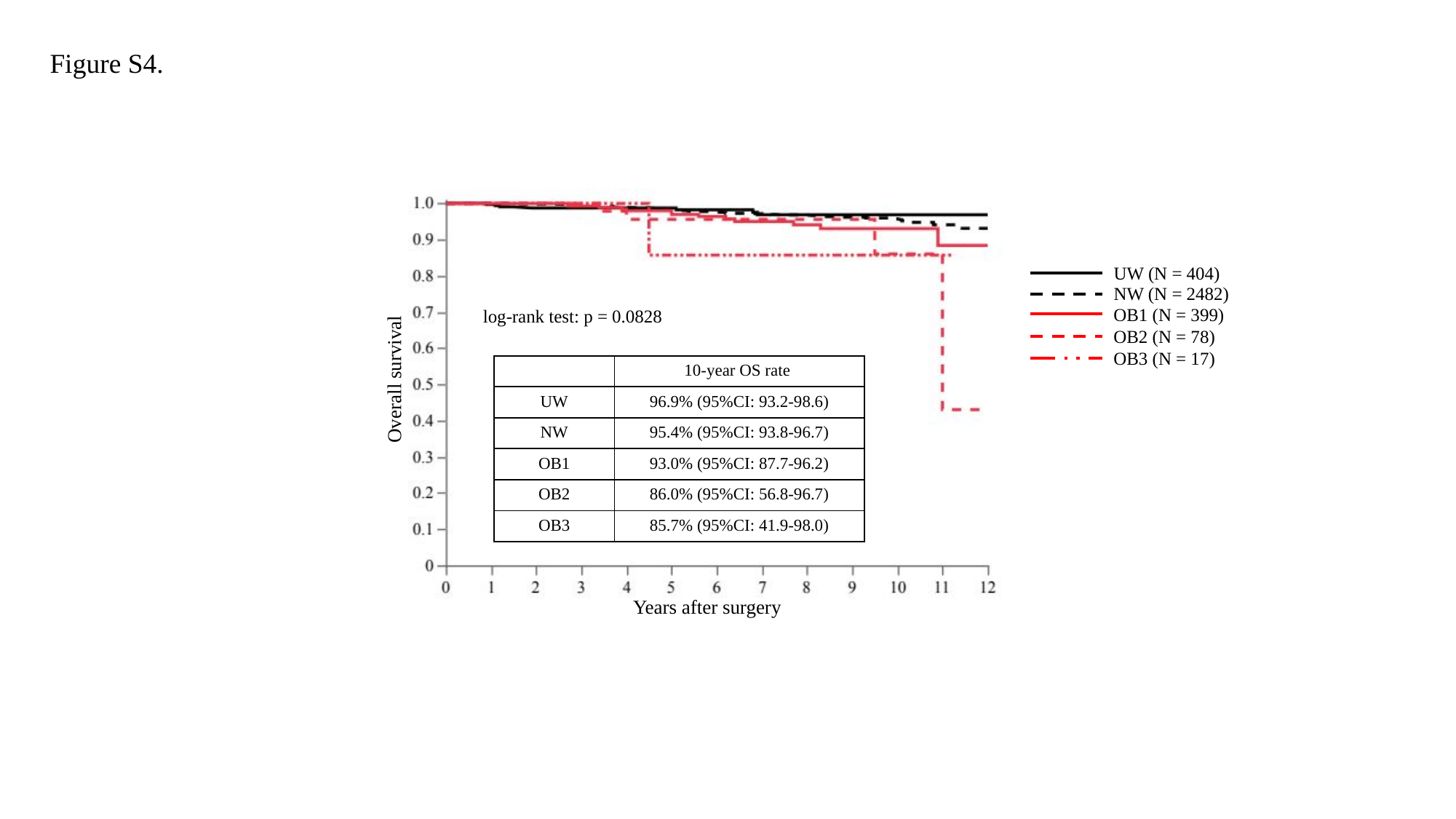

Figure S4.
UW (N = 404)
NW (N = 2482)
OB1 (N = 399)
OB2 (N = 78)
OB3 (N = 17)
log-rank test: p = 0.0828
| | 10-year OS rate |
| --- | --- |
| UW | 96.9% (95%CI: 93.2-98.6) |
| NW | 95.4% (95%CI: 93.8-96.7) |
| OB1 | 93.0% (95%CI: 87.7-96.2) |
| OB2 | 86.0% (95%CI: 56.8-96.7) |
| OB3 | 85.7% (95%CI: 41.9-98.0) |
Overall survival
Years after surgery
