# Supplementary material for: The impact of obesity and endocrine therapy on the prognosis of premenopausal women with hormone receptor‐positive breast cancer: A single‐institute retrospective study
Source: Cancer Rep (Hoboken). 2022 Aug 9;6(2):e1695. doi: 10.1002/cnr2.1695 (PMC9940008; doi:10.1002/cnr2.1695)
Supplement: Supplementary file 2 — TABLE S1 Multivariate analysis of prognostic factors for BCSS in NW and OB3 premenopausal patients with stage II/III breast cancer TABLE S2. Multivariate analysis of prognostic factors for OS in premenopausal patients [file CNR2-6-e1695-s001.docx]

**Table S1. Multivariate analysis of prognostic factors for BCSS in NW and OB3 premenopausal patients with stage II/III breast cancer**

| **Prognostic factor** | **Adjusted HR** | **95% confidential interval** | **p-value** |
| --- | --- | --- | --- |
| OB3 vs. NW | 10.88 | 2.57-46.1 | 0.0162 |
| Ki67 int./high vs. low | 2.38 | 0.29-19.43 | 0.4993 |
| Lymph node-positive vs. negative | 2.36 | 1.21-4.61 | 0.0084 |

Abbreviations: BCSS, breast cancer specific survival; UW, underweight; NW, normal weight; OB1, obese 1 degree; OB2, obese 2 degree; OB3, obese 3 degree; HR, hazard ratio

**Table S2. Multivariate analysis of prognostic factors for OS in premenopausal patients**

| **Prognostic factor** | **Adjusted HR** | **95% confidential interval** | **p-value** |
| --- | --- | --- | --- |
| OB1-3 vs. UW/NW | 0.93 | 0.32-2.69 | 0.8895 |
| stage II/III vs. I | 1.57 | 0.70-3.50 | 0.2728 |
| NG 2/3 vs. 1 | 2.20 | 0.86-5.59 | 0.0988 |
| Ki67 int./high vs. low | 0.49 | 0.14-1.72 | 0.2658 |
| Chemotherapy yes vs. no | 1.63 | 0.71-3.69 | 0.2453 |

Abbreviations: OS, overall survival; UW, underweight; NW, normal weight; OB1, obese 1 degree; OB2, obese 2 degree; OB3, obese 3 degree; HR, hazard ratio; NG, nuclear grade

**Figure Legends**

**Figure S1: Breast cancer specific survival in OB1-3 premenopausal patients**

Abbreviations: BCSS, breast cancer specific survival; UW, underweight; NW, normal weight; OB1, obese 1 degree; OB2, obese 2 degree; OB3, obese 3 degree; HR, hazard ratio; TAM, tamoxifen; OFS, ovarian function suppression; CI, confidence interval

**Figure S2: Breast cancer specific survival in OB1-3 patients with a propensity matching model**

Abbreviations: OFS, ovarian function suppression; CI, confidence interval

**Figure S3: Breast cancer specific survival by BMI (UW, NW, OB1, OB2 and OB3)**

Abbreviations: BMI, body mass index; BCSS, breast cancer specific survival; UW, underweight; NW, normal weight; OB1, obese 1 degree; OB2, obese 2 degree; OB3, obese 3 degree; CI, confidence interval

**Figure S4. Overall survival by BMI (UW, NW, OB1, OB2 and OB3)**

Abbreviations: BMI, body mass index; OS, overall survival; UW, underweight; NW, normal weight; OB1, obese 1 degree; OB2, obese 2 degree; OB3, obese 3 degree; CI, confidence interval
